# Total tumor volume reduction and low PSMA expression in patients receiving Lu-PSMA therapy

**DOI:** 10.7150/thno.60222

**Published:** 2021-07-13

**Authors:** Robert Seifert, Katharina Kessel, Katrin Schlack, Matthias Weckesser, David Kersting, Konstantin E. Seitzer, Manuel Weber, Martin Bögemann, Kambiz Rahbar

**Affiliations:** 1 Department of Nuclear Medicine, University Hospital Essen, Essen, Germany; 2 Department of Nuclear Medicine, University Hospital Münster, Münster, Germany; 3 German Cancer Consortium (DKTK); 4 West German Cancer Center; 5 Department of Urology, University Hospital Münster, Münster, Germany

**Keywords:** ^177^Lu-PSMA-617, mCRPC, radioligand therapy

## Abstract

**Background:** [^177^Lu]-PSMA-617 (Lu-PSMA) therapy is a promising therapeutic option for end-stage prostate cancer patients. Early treatment response at the first restaging after two therapy cycles might correlate with high treatment efficacy and long overall survival (OS). Therefore, the aim of this study was to evaluate whether early reduction in tumor volume is a positive prognosticator for OS. To this end, PSMA PET prior to therapy (baseline) and at first restaging after two therapy cycles (interim; i.e., 12 weeks) were compared.

**Methods:** Patients with metastatic castration-resistant prostate cancer who received Lu-PSMA therapy were reviewed for this analysis. All patients with available baseline and interim [^68^Ga]-PSMA-11 PET/CT were included in this analysis (n = 33). All PSMA avid metastases in baseline and interim PETs were semi-automatically segmented. The average PSMA expression (mean SUV_max_ of all metastases), total tumor volume (PSMA-TV) and TLQ (quotients of tumor volume and SUV_mean_ summed over all metastases) were quantified at baseline and interim timepoints. Response in PSMA-TV was assumed when a decline > 30% was present. OS and biochemical response were available for all patients.

**Results:** Baseline PSMA-TV was a statistically significant prognosticator of OS (HR = 1.618 95%CI: 1.117 - 2.343, p = 0.011). Reduction in PSMA-TV was not a statistically significant positive prognosticator of OS in the total cohort (HR = 0.829 95%CI: 0.559 - 1.230, p = 0.352). Likewise, there was no statistical difference in survival time comparing patients with PSMA-TV response to those without (13.2 *vs.* 15.6 months, p = 0.1). In the subgroup of patients with PSMA-TV response, mean SUV_max_ was a statistically significant prognosticator of OS (binarized by median; HR = 0.15; 95%CI: 0.03 - 0.83; p < 0.05). If patients with low PSMA expression at baseline were excluded from the analysis, reduction in PSMA-TV became a positive prognosticator of OS in uni- and multivariable Cox regression (HR = 0.290; 95%CI: 0.108 - 0.782; p = 0.015).

**Conclusion:** PSMA-TV reduction was a positive prognosticator of OS only if patients with low PSMA expression were excluded. This might indicate that the PSMA-PETs of patients with low PSMA expression may not be suited for assessing PSMA-TV reduction. Future studies investigating the interplay of PSMA-TV and low PSMA expression response are warranted.

## Introduction

Patients with metastasized castration-resistant prostate cancer (mCRPC) ultimately undergo progression and most likely have a fatal outcome 1. However, [^177^Lu]Lutetium PSMA-617 (Lu-PSMA) radioligand therapy or similar ligands like PSMA I&T that target the prostate-specific membrane antigen (PSMA) are showing high biochemical response rates (up to 66%) and have a favorable toxicity profile in mCRPC patients 2-8. Yet, the monitoring of treatment response to Lu-PSMA therapy and outcome prognostication is a clinical issue that has to date been inadequately addressed.

Recently, PSMA targeting positron emission tomography (PET) criteria have been proposed to assess the treatment responses of patients with mCRPC 9, 10. These criteria recommend the integration of PSMA PET derived total tumor volume as a metric to monitor the course of the disease. We and others could show that baseline PSMA PET derived tumor volume quantification is feasible and that pretherapeutic tumor volume is a statistically significant prognosticator of outcome in patients treated with Lu-PSMA therapy 11-13.

Preliminary data suggests that PSMA PET derived total tumor volume (PSMA-TV) changes can be employed to monitor treatment response to Lu-PSMA therapy 14, 15. However, *Schmuck et al.* only included ten patients for tumor volume monitoring under Lu-PSMA therapy and *Grubmüller et al.* employed an unusual interim PET timepoint after the third administration of Lu-PSMA therapy 14, 15. Therefore, a comprehensive analysis of the implications of PSMA-TV changes between baseline and interim PET after two cycles of Lu-PSMA therapy is still missing.

Early decrease of PSMA-TV may indicate response, but could also be caused by low PSMA expression due to cancer cell dedifferentiation and thus be associated with short OS. We showed previously that initially low PSMA expression may be a statistically significant negative prognosticator of overall survival (OS) 16. However, the interplay of PSMA-TV reduction and PSMA expression is poorly elucidated to date.

Therefore, the aim of the present study was to compare baseline and interim PSMA PET derived PSMA-TV to assess whether prolonged OS is associated with PSMA-TV response. Additionally, the PSMA expression of patients with PSMA-TV response was investigated.

## Methods

### Patients

All patients who received Lu-PSMA therapy in the Department of Nuclear Medicine of the University Hospital Münster were selected for this study if baseline and interim PSMA-11 PET CT were present. A total number of 33 patients met these inclusion criteria. Detailed patient characteristic data is provided in Table 1.

### PSMA PET acquisition

PSMA PET imaging and tracer synthesis was done as previously reported 11. Briefly, [^68^Ga]Gallium-PSMA-11 was employed for PET imaging. The PSMA-11 precursor was delivered by ABX (ABX GmbH, Radeberg, Germany). Image acquisition started 60 minutes after injection using a Biograph mCT (Siemens Healthineers, Knoxville, TN, United States). The PET scan area included the vertex to thigh. A mean dose of 163 (standard deviation: 24) MBq was administered. Whole-body low dose and abdominal full dose (contrast-enhanced) PET acquisitions were obtained.

### PSMA therapy

Prostate cancer patients referred to the Department of Nuclear Medicine Münster for Lu-PSMA therapy were treated with PSMA-617 (ABX advanced biochemical compounds, Radeberg, Germany) conjugated with [^177^Lu]Lutetium (ITG Isotopes Technology, Garching, Germany). The Lu-PSMA therapy was administered at a 6-8 weeks interval.

### Image interpretation

Sixty-six PSMA PET CTs were semi-automatically analyzed as previously published (33 baseline and 33 interim PETs) 11. The software research prototype MICIIS was employed for image analysis (formerly MI Whole Body Analysis Suite, MIWBAS, Siemens Healthineers, Knoxville, TN, United States). Briefly, all foci with increased PSMA uptake were automatically segmented employing a liver-specific threshold, which leads to the segmentation of physiological and pathological foci (i.e. all lesions with an SUV_max_ greater than the liver threshold were segmented). The liver-specific segmentation threshold was automatically determined (threshold = (4.3 / liver SUV_mean_) × (liver SUV_mean_ + liver SUV_standard deviation_). Tracer foci caused by physiological uptake were semi-automatically removed from the analysis: As previously published, foci within the kidney, spleen and liver can be automatically removed. A trained nuclear medicine physician then manually checked the preliminary segmentation and adjusted the result if needed. Adjustments typically included the re-segmentation of liver metastases and the exclusion of gastrointestinal tracer uptake. Thereby, only metastases were segmented ultimately. For each selected focus, 50% of the local SUV_max_ was used to segment the metastasis. TLQ_lesion_ (total lesion quotient) was defined as the quotient of metabolic tumor volume and the SUV_mean_ of a given focus as previously reported 12. For each metastasis, SUV_max_, TLQ_lesion_ and metabolic tumor volume were noted. For each PSMA PET acquisition, the metrics of all metastases were summed to obtain the total tumor volume (PSMA-TV) and TLQ metric, or averaged to obtain mean SUV_max_. Volumetric response was defined as a decline greater than 30% comparing baseline and interim PSMA PET, as previously published by Grubmüller *et al.* 17. Additionally, the 30% decline criterion is also employed by RECIST and PERCIST and seems to be justified by SUV repeatability measurements of PSMA PET 18-20.

### Statistical analysis

R (version 4.0.3) was used for descriptive analysis, Wilcoxon test, Spearman correlation, Cox regression, Martingale analysis and plotting 21. For the cutoff finding of continuous variables, the R package “maxstat” was used. For Cox regression and Martingale analysis, response in PSMA-TV was expressed as a ratio (PSMA‑TV_baseline_ / PSMA‑TV_interim_). All parameters were log transformed for Cox regression and Martingale analysis. P < 0.05 was used as the significance level.

## Results

### Patient characteristics at baseline and interim time point

Detailed patient characteristics are provided in Table 1. The baseline median PSMA-TV was 138 ml (IQR = 230 ml), the interim median PSMA-TV was 107 ml (IQR = 199 ml). The median OS of the entire cohort was 15 (8.6 - 25.9) months.

### Association of baseline PSMA PET derived parameters and overall survival

Baseline PSMA-TV was a statistically significant negative prognosticator of OS in univariate Cox regression (HR = 1.618 95%CI: 1.117 - 2.343, p = 0.011). Baseline mean SUV_max_ was not a statistically significant prognosticator of OS in univariate Cox regression (HR = 0.416 95%CI: 0.133 - 1.30, p = 0.131). Multivariable regressions of baseline PET derived parameters (PSMA-TV, TLQ and mean SUV_max_) adjusted for baseline blood tumor markers LDH and PSA are shown in Table 2. Only baseline TLQ remained a statistically significant prognosticator of OS in a multivariable regression adjusted for PSA and LDH levels (HR = 2.33; 95%CI: 1.23-4.42; p = 0.009). Supplemental Figure 1 shows a Martingale residual analysis.

**Figure 1:**
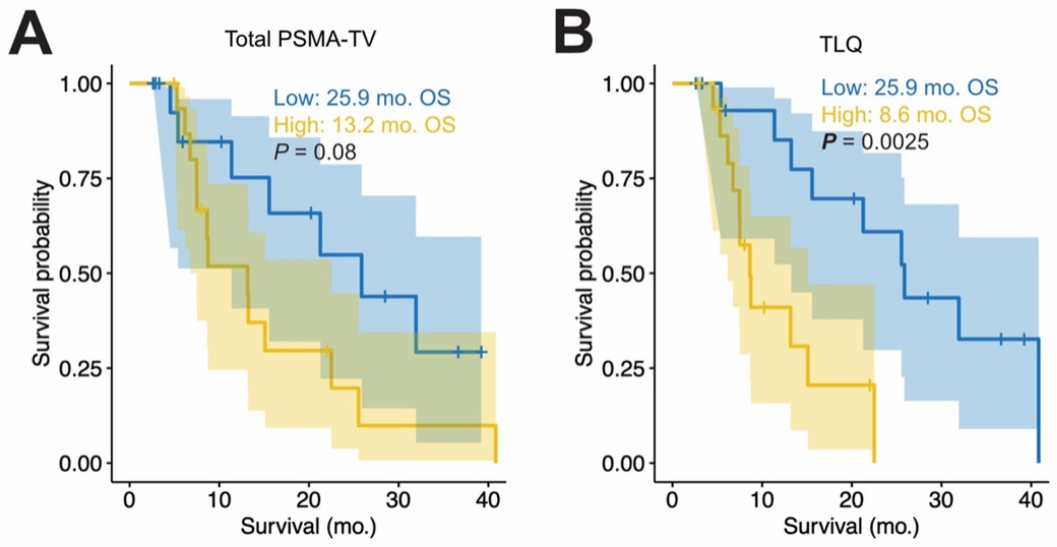
** Baseline PET metrics and overall survival.** Kaplan Meier plots of OS are shown for the total tumor volume (PSMA-TV, A) and the summed TLQ score (TLQ = quotient of tumor volume and SUV_mean_ of all metastases are summed, B). Median OS in months (mo) and log rank p values are shown additionally. For all panels, the median of the PET measurements was employed to distinguish between patients with high (yellow) and low (blue) values.

To visualize the OS, the medians of baseline PSMA-TV and TLQ were used to group the patients with high or low PET metrics; Figure 1 shows the OS by Kaplan Meier plots. Patients with high PSMA-TV did not have shorter median OS compared to those with low tumor volume (13.2 *vs.* 25.9 months, p = 0.08; HR = 2.314, 95%CI: 0.885 - 6.049, p = 0.087). In contrast, patients with high mean SUV_max_ had longer OS compared to those without (22.5 *vs.* 11.4 months, p = 0.023; HR = 0.352, 95%CI: 0.138 - 0.899, p = 0.029).

### Association of interim PSMA PET derived parameters and OS

Interim PSMA-TV was a statistically significant prognosticator of OS in univariate Cox regression (HR = 1.427 95%CI: 1.06 - 1.921, p = 0.019). Interim mean SUV_max_ was not a statistically significant prognosticator of OS in univariate Cox regression (HR = 0.963, 95%CI = 0.519 - 1.786, p = 0.904).

### Association of total tumor volume (PSMA-TV) response with PSA response

**Figure 2:**
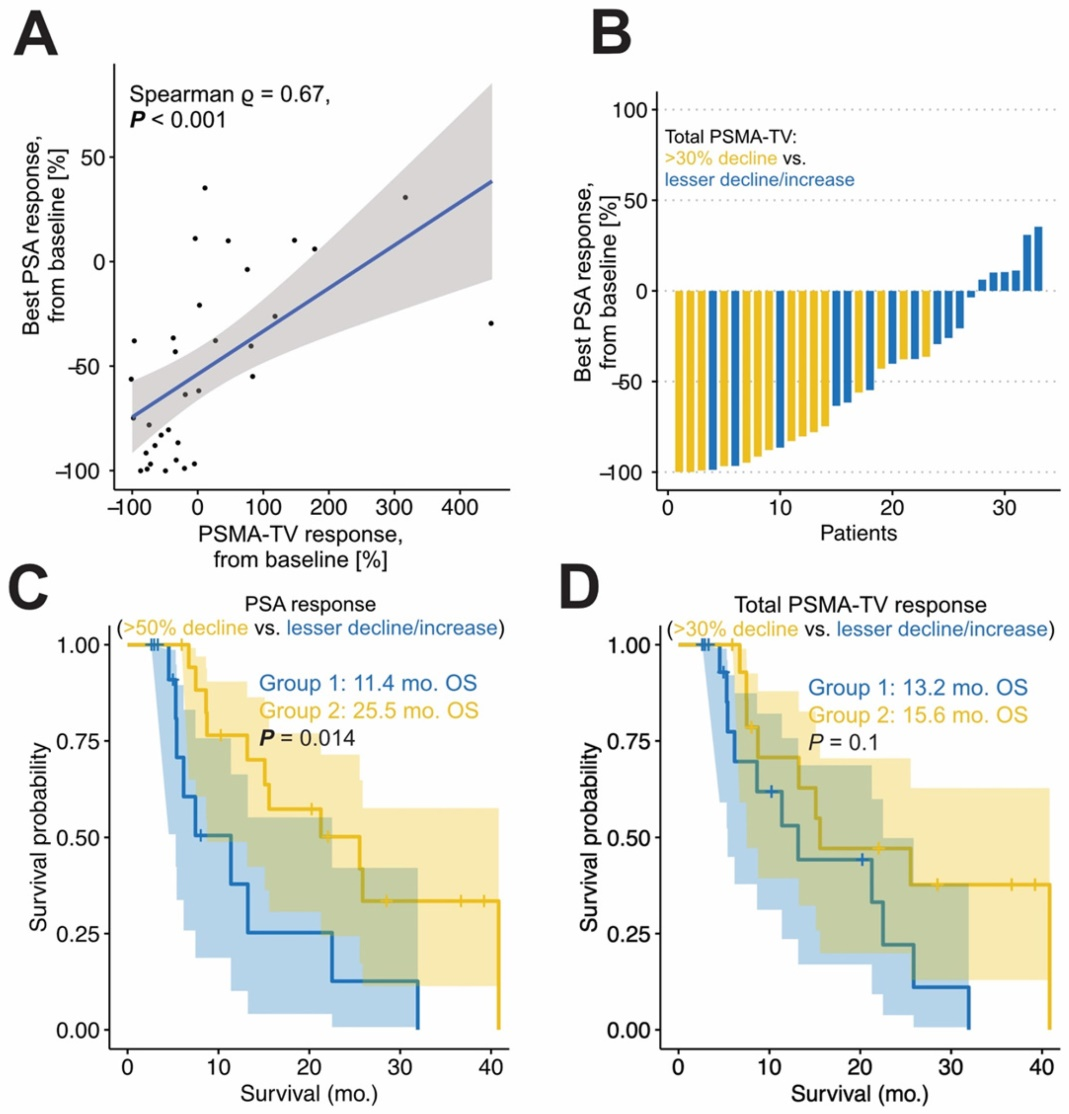
** Response in the total tumor volume.** The correlation of response in total tumor volume (PSMA-TV) and best PSA response is shown in panel A. The Waterfall plot (B) shows best PSA decline of the included cohort, yellow patients had a PSMA-TV decline of >30 %, whereas blue patients had not. Patients with PSA response have a significantly longer overall survival (C). Patients with a total tumor volume decline of greater 30% (Group 2, D) did not have a significantly longer OS compared to patients without (Group 1).

A total number of 15 patients had a PSMA-TV tumor decline greater than 30%. The best relative PSA response and relative PSMA-TV_response_ were significantly correlated (Spearman ρ = 0.67; p < 0.001). Patients with biochemical response had significantly greater PSMA-TV decline compared to patients without biochemical response (-51% *vs.* 48%, p < 0.001). Additionally, patients with PSA response had significantly longer OS (25.5 *vs.* 11.4 months p < 0.05). See Figure 2C for details.

### Association of total tumor volume (PSMA-TV) response with OS

PSMA-TV_response_ was not a statistically significant prognosticator of OS in univariate Cox regression (HR = 0.829 95%CI: 0.559 - 1.230, p = 0.352). Multivariable regression is shown in Table 3. PSMA-TV_response_ was not a statistically significant prognosticator for OS in a multivariable regression adjusted for PSA and LDH (p = 0.430). Martingale residual analysis is shown in supplemental Figure 1. Confirming expectations, patients with a tumor volume reduction did not have a significantly longer OS compared to those without (15.6 *vs.* 13.2 months, p = 0.1; HR = 0.475, 95%CI: 0.190 - 1.189, p = 0.112; see Figure 2D).

### Association of total tumor volume (PSMA-TV) response and OS in patients without low PSMA expression

**Figure 3:**
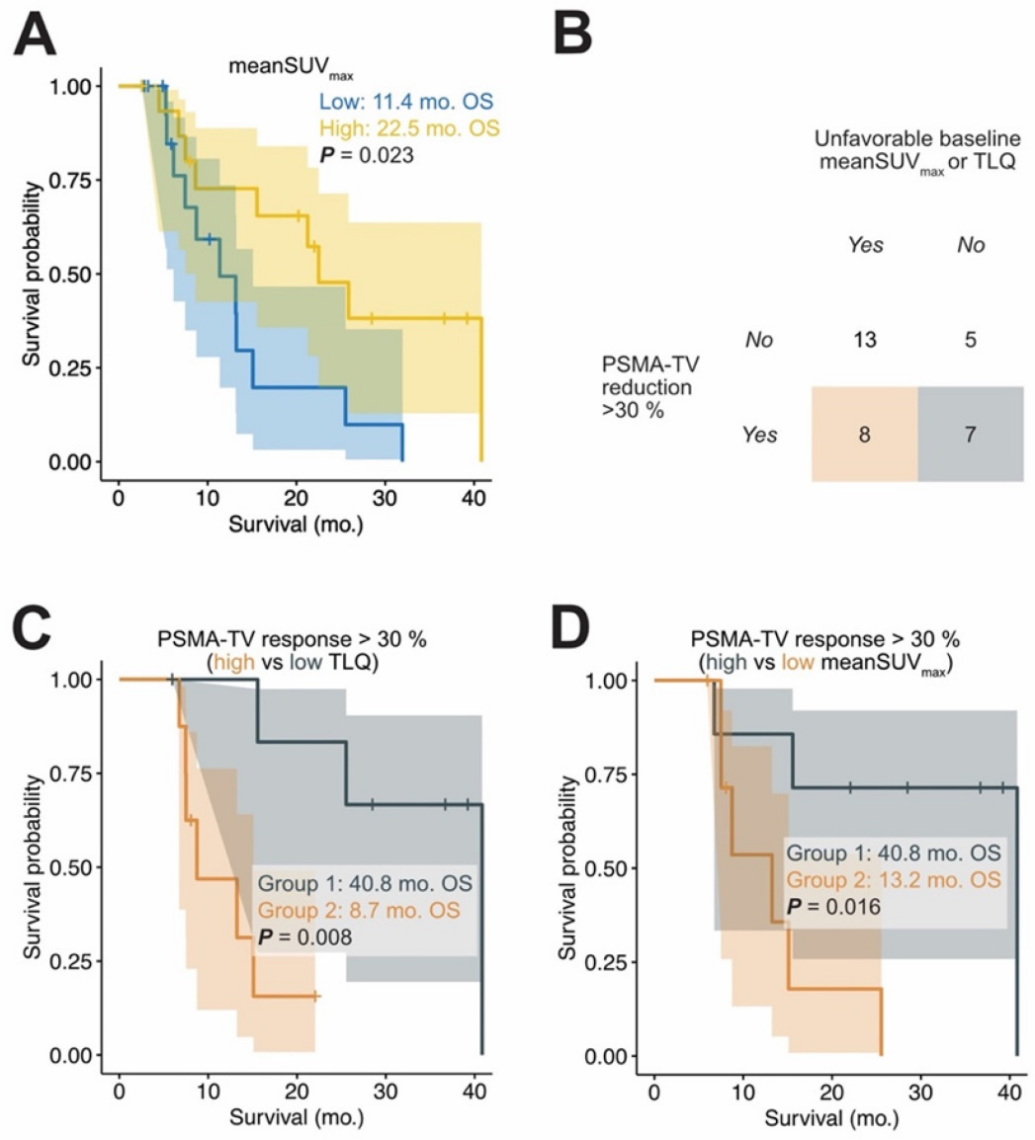
** Divergence between tumor volume response and baseline stratification.** Patients with low baseline meanSUV_max_ have a significantly shorter OS time (A). The median was used to seperate between patients with high (yellow) and low (blue) meanSUV_max_. A considerable fraction of patients with tumor volume response greater than 30% had a high baseline TLQ and low meanSUVmax (B). Patients with a total tumor volume response of >30% were further stratified by low/high baseline TLQ (C) and low/high baseline meanSUV_max_ (D). To this end, the meanSUV_max_ and TLQ cutoffs to distinguish between patients with low and high values were determined by a log-rank cutoff finder (meanSUV_max_ threshold = 22.8; TLQ threshold = 34.4); this binarization was only done for visualization purposes. Despite presence of volumetric tumor response, baseline TLQ and meanSUV_max_ identified patients at risk of poor outcome.

In total, 27 out of 33 patients had high mean SUV_max_ expression at baseline according to the previously published threshold (high PSMA expression: mean SUV_max_ > 14.3). Paradoxically, 20% of all PSMA-TV responding patients had low mean SUV_max_ expression at baseline, which is a known negative prognosticator of OS (Figure 3) 12, 16. Table 4 shows the characteristics of PSMA-TV responding patients. In patients with PSMA-TV response, baseline mean SUV_max_ was a statistically significant prognosticator of OS (binarized by median; HR = 0.15; 95%CI: 0.03-0.83; p < 0.05). To investigate whether patients with low mean SUV_max_ influenced the prognostication of OS by exhibiting strong PSMA-TV reduction, patients with low mean SUV_max_ were excluded from the analysis. In the subgroup of patients without low PSMA expression (mean SUV_max_ > 14.3 according to *Seifert et al.*), the reduction of PSMA-TV was a statistically significant positive prognosticator of OS (HR = 0.378 95%CI: 0.169 - 0.848, p = 0.018) 16. Moreover, PSMA-TV response remained a significant prognosticator in a multivariable analysis adjusted for baseline PSA and LDH levels (HR = 0.290 95%CI: 0.108 - 0.782, p = 0.015).

## Discussion

The reduction in PSMA PET derived total tumor volume (PSMA-TV) in response to [^177^Lu]Lutetium-PSMA617 therapy was evaluated as a prognosticator for OS in the present study. To this end, the pretherapeutic (baseline) and interim PSMA PET/CTs after two cycles of Lu-PSMA therapy were compared. If the entire cohort was regarded, PSMA-TV reduction was surprisingly not a relevant prognosticator of OS. If patients with low PSMA expression were excluded from the analysis, PSMA-TV reduction was a statistically significant positive prognosticator of OS. Therefore, reduction of PSMA-TV appears to be a valuable parameter to prognosticate OS, but the clinician must carefully consider PSMA expression to avoid misleading interpretations of PSMA-TV reductions.

Lu-PSMA therapy is a promising therapeutic option for patients with advanced prostate cancer and has been evaluated in several retrospective and prospective studies 2, 7, 22. However, the identification of patients who respond to Lu-PSMA is a clinical issue that has not been sufficiently addressed. Especially with the rise of other targeted therapeutics like Olaparib that might be alternatives to Lu-PSMA therapy, the long-term prediction of response and prognostication of outcome for patients prior to Lu-PSMA therapy is gaining importance 23. Recently, the utilization of PSMA-TV has been proposed as a biomarker to assess therapy response 9. Despite preliminary studies that identified the reduction in PSMA-TV as a prognosticator of OS, the interplay of PSMA-TV with the degree of PSMA expression is still poorly elucidated. Moreover, previously published studies have either suffered from small patient cohorts or a limited number of events (only 11 deaths out of 38 patients) 14, 15.

The present study indicates that baseline PSMA-TV is a statistically significant prognosticator of OS in patients treated with Lu-PSMA therapy. Surprisingly, the decrease of PSMA-TV was not a statistically significant prognosticator in the present cohort. To investigate these counterintuitive findings, the baseline PSMA expression of patients with tumor volume response and short overall survival was analyzed. We hypothesized that the reduction of PSMA-TV cannot be regarded without looking at the PSMA expression. The reason for this is that in advanced prostate cancer, cancer cells might undergo dedifferentiation and lose the ability to express PSMA 24, 25. Therefore, PSMA-TV could be biased by the presence of unsegmentable metastases with faint PSMA expression.

**Figure 4:**
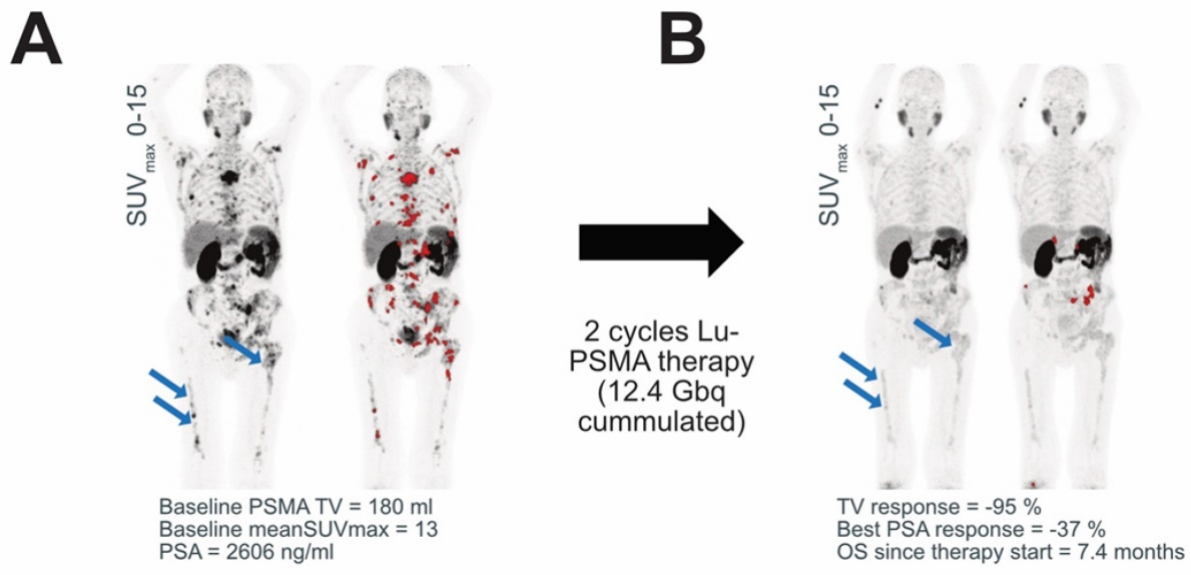
** PSMA expression.** An exemplary patient is shown before (A) and after (B) Lu-PSMA therapy. The patient had a low baseline PSMA expression and high TLQ. The MIP of the PSMA -PET acquisition is shown with and without segmented metastases (marked in red). After Lu-PSMA therapy, the PSMA expression decreased, and the diffuse bone marrow involvement (blue arrows) has only minimal PSMA uptake. Therefore, less tumor volume is segmented, which erroneously suggested PSMA-TV response.

The reduction of PSMA‑TV was a statistically significant prognosticator of OS in the present study in patients without low PSMA expression. This indicates that the reduction of PSMA‑TV in the interim PET can be erroneously interpreted as a treatment response in the presence of metastases with low PSMA expression at baseline (Figure 4). Patients with low baseline PSMA expression do not seem to be suited for interim PET tumor volumetry and PSMA-TV reduction quantification. Therefore, PSMA expression should be checked when the reduction of PSMA-TV in response to Lu-PSMA therapy is assessed. Future studies should evaluate whether PSMA-TV only captures metastases with sufficient PSMA uptake for Lu-PSMA therapy but neglects metastases with insufficient PSMA uptake.

Patients with a biochemical response (PSA decline greater than 50%) had a longer OS compared to patients without. This seems contradictory to the finding that patients with tumor volume response did not show a longer OS in the total cohort. However, the PSA value might more robustly quantify the tumor volume and may not be influenced by metastases with low PSMA expression. It must be noted that different PSMA-TV response thresholds than the commonly employed 30% decline might identify patients with short overall survival. However, given the reproducibility of PSMA PET metrics in the range of +/- 30%, only the 30 % cutoff was used for PSMA-TV in the present study 20.

The present study faces some limitations. There might be a selection bias, as only patients with two cycles of Lu-PSMA therapy were included. The reason for this is because restaging after the start of therapy is generally performed after the administration of the second cycle of Lu-PSMA therapy. Patients with rapid progression of prostate cancer, who might therefore not have received a second cycle of Lu-PSMA therapy, were not included. This might impair the transferability of the results to larger patient cohorts. Additionally, the included patient cohort is still relatively small. Because we only included patients who received PSMA-11 PET/CT at baseline and follow-up, patients who received combinations of PSMA-11 and PSMA-1007 PET CTs were not included in this study. However, given the high rate of events (60%), we feel that this limitation should not impair the findings of this study. A limitation of the PSMA PET volumetry is that only metastases with elevated PSMA expression are captured. Due to dedifferentiation and decreasing PSMA expression, the tumor volume might erroneously appear regredient, while the actual tumor volume is progredient. The contrary report of *Grubmüller et al.*, who found PSMA-TV reduction to be associated with longer OS, might be partly explained by the more advanced cancer stages in our cohort (median LDH = 282 *vs.* 189 U/L; median PSA 146 *vs.* 61 ng/mL), which could correlate with a larger fraction of patients who show low PSMA expression 15. Therefore, future studies should find new methods to measure the whole-body tumor volume in patients with low PSMA expression. Finally, we did not evaluate the occurrence of new metastases as extra category, which per se leads to a progredient disease rating. This contrasts with RECIST, in which the occurrence of new metastases automatically leads to progredient disease. However, RECIST cannot measure a total tumor volume but relies on exemplary lesions. Therefore, the new metastases criterion is needed to assess the disease status in RECIST. Future studies are needed to evaluate relevance of new metastases in the context of whole-body tumor volume measurements.

## Conclusion

Decline in PSMA PET derived total tumor volume is not a prognosticator of OS in patients treated with Lu-PSMA therapy, if patients with low PSMA expression were included in the cohort. If patients with low PSMA expression were excluded, reduction in PSMA-TV is a significant prognosticator of OS, even in multivariable analysis. Therefore, PSMA-TV alone is not suited to monitor the progression of disease in end-stage prostate cancer patients. This might be due to erroneously quantified interim tumor volume reduction in the event of low PSMA uptake. Further studies focusing on the tumor volume response of patients with advanced prostate cancer are warranted. Tumor volume reduction should be carefully regarded in conjunction with baseline PSMA expression to properly assess treatment response.

## Supplementary Material

Supplementary figure.

## Figures and Tables

**Table 1:** Patient characteristics

Patient characteristics	Total cohort	TV response >30%	TV response =<30%
Number of patients	33	15	18
Age	73.1 [10.7]	74.9 [9.5]	68.3 [13.0]
Previous therapies			
Docetaxel	25 (75.8%)	11 (73%)	14 (77%)
Cabazitaxel	5 (15.2%)	1 (6.7%)	4 (22.2%)
Abiraterone	22 (66.7%)	6 (40.0%)	13 (72.2%)
Enzalutamide	17 (51.5%)	10 (66.7%)	7 (38.9%)
Blood parameter before Lu-PSMA			
Aspartate aminotransferase [U/l]	29.0 [13.0]	29.0 [17.0]	28.5 [11.5]
Alanine transaminase [U/l]	17.0 [11.0]	17.0 [12.0]	15.5 [9.3]
White blood cell count [/µl]	6.2 [2.4]	6.0 [2.4]	6.5 [2.6]
Hemoglobin [g/dl]	11.4 [2.5]	11.5 [1.5]	11.3 [3.3]
Platelets [/µl]	247 [102]	200 [101]	246 [84]
Prostate-specific antigen [ng/ml]	146.1 [344.9]	284.0 [641.6]	122.3 [254.2]
Lactate dehydrogenase [U/l]	282.0 [159.0]	282.0 [114.5]	252.0 [219.0]
Lu-PSMA therapy			
Number of Lu-PSMA cycles	4 4	6 [3.5]	3.5 [3.5]
Average activity per cycle [GBq]	6.2 [1.1]	6.2 [0.3]	6.4 [1.2]
Cumulated activity [GBq]	30.7 [27.8]	38.1 [22.4]	22.9 [27.4]
PSMA PET			
Days between baseline PET and first cycle [days]	26 [17.0]	26 [18.5]	29 [16.5]
Days between second cycle and interim PET [days]	42 [17.0]	43 [17.0]	39 [17.3]
Days elapsed between interim and baseline PET [days]	133 [41]	134 [45.5]	130 [37.5]
Baseline PSMA-TV	138.2 [229.7]	184.2 [237.7]	129.9 [181.0]
Metastatic spread			
Liver metastases	9 (27.3%)	6 (40.0%)	3 (16.7%)
Bone metastases	28 (84.8%)	13 (86.7%)	15 (83.3%)
Lymph nodes metastases	30 (90.9%)	13 (86.7%)	17 (94.4)
Lung metastases	6 (18.2%)	3 (20%)	3 (16.7%)
Brain metastases	0 (0%)	0 (0%)	0 (0%)

TV = PSMA PET derives total tumor volume; Lu-PSMA = [^177^Lu]Lutetium-PSMA-617 therapy. Median or frequency is reported. Interquartile range is given in brackets. Relative frequency is given in parentheses. TV response is defined as a decline in TV greater than 30% comparing pretherapeutic baseline and interim PET after two cycles of Lu-PSMA therapy.

**Table 2:** Baseline PSMA PET derived parameters adjusted for baseline PSA and LDH in multivariable Cox regression models.

Parameter	Hazard ratio	95% CI of Hazard ratio	*P* value of covariate	LR ratio test *P* value
PSMA-TV	1.50	0.85 - 2.63	0.159	0.05
* PSA*	1.06	0.70 - 1.62	0.770	
* LDH*	1.18	0.31 - 4.49	0.806	
TLQ	2.33	1.23 - 4.42	**0.009**	**0.004**
* PSA*	0.99	0.67 - 1.45	0.940	
* LDH*	0.82	0.22 - 3.04	0.771	
meanSUV_max_	0.35	0.10 - 1.29	0.117	**0.04**
* PSA*	1.40	1.03 - 1.90	**0.030**	
* LDH*	0.96	0.22 - 4.30	0.965	

N = 32 due to availability of data. CI = Confidence interval; LR = likelihood ratio. Parameters were log transformed for regression. See methods section for details.

**Table 3:** Response in PSMA-TV adjusted for baseline PSA and LDH in multivariable Cox regression models.

Cohort	Parameter	Hazard ratio	95% CI of Hazard ratio	*P* value of covariate	LR ratio test *P* value
Entire cohort (n=32)	PSMA-TV_response_	0.843	0.55 - 1.29	0.430	0.09
	* PSA*	1.33	0.99 - 1.77	0.056	
	* LDH*	1.28	0.39 - 4.239	0.689	
Patients without low PSMA expression (n=27)	PSMA-TV_response_	0.290	0.108 - 0.782	**0.015**	**0.008**
	* PSA*	1.402	0.995 - 1.976	0.053	
	* LDH*	1.163	0.342 - 3.962	0.808	

N = 32 due to availability of data. CI = Confidence interval; LR = likelihood ratio. Parameters were log transformed for regression. No low PSMA expression was defined as meanSUVmax > 14.3. See methods section for details.

**Table 4:** Characteristics of patients with PSMA-TV response separately for the levels of PSMA expression.

Patient characteristics	Total subgroup (TV response >30%)	Low PSMA expression	No low PSMA expression	P value
Number of patients	15	3	12	
Age	74.9 [9.5]	77.0 [1.6]	73.5 [10.9]	0.145
Previous therapies				
Docetaxel	11 (73%)	1 (33.3%)	10 (83.3%)	0.08
Cabazitaxel	1 (6.7%)	0 (0.0%)	1 (8.3%)	0.605
Abiraterone	6 (40.0%)	2 (66.7%)	7 (58.3%)	0.792
Enzalutamide	10 (66.7%)	2 (66.7%)	8 (66.7%)	1.000
Blood parameter before Lu-PSMA				
Prostate-specific antigen [ng/ml]	284.0 [641.6]	40.5 [1296.5]	289.3 [573.7]	0.54
Lactate dehydrogenase [U/l]	282.0 [114.5]	282.0 [139.5]	288.0 [101.8]	0.927
Metastatic spread				
Liver metastases	6 (40.0%)	2 (66.7%)	4 (33.3%)	0.292
Bone metastases	13 (86.7%)	3 (100.0%)	10 (83.3%)	0.448
Lymph nodes metastases	13 (86.7%)	2 (66.7%)	11 (91.7%)	0.255
Lung metastases	3 (20%)	1 (33.3%)	2 (16.7%)	0.519

TV = PSMA PET derives total tumor volume. Median or frequency is reported. Interquartile range is given in brackets. Relative frequency is given in parentheses. No low PSMA Expression is defined as meanSUV_max_ > 14.3. T-test and chi-square were used to compare groups.

## Abbreviations

TV: Tumor Volume
PSMA: Prostate Specific Membrane Antigen

## References

[1] Sartor O, de Bono JS (2018). Metastatic Prostate Cancer. Longo DL, Ed. N Engl J Med.

[2] Rahbar K, Ahmadzadehfar H, Kratochwil C (2017). German Multicenter Study Investigating 177Lu-PSMA-617 Radioligand Therapy in Advanced Prostate Cancer Patients. J Nucl Med.

[3] Seifert R, Kessel K, Schlack K, Weckesser M, Bögemann M, Rahbar K Radioligand therapy using [177Lu]Lu-PSMA-617 in mCRPC: a pre-VISION single-center analysis. Eur J Nucl Med Mol Imaging. 2020.

[4] Kessel K, Seifert R, Schäfers M (2019). Second line chemotherapy and visceral metastases are associated with poor survival in patients with mCRPC receiving 177 Lu-PSMA-617. Theranostics.

[5] Seifert R, Kessel K, Boegemann M (2019). Additional local therapy of liver metastases in mCRPC patients receiving systemic PSMA targeted therapy. J Nucl Med.

[6] Hofman MS, Emmett L, Sandhu S (2021). [177Lu]Lu-PSMA-617 versus cabazitaxel in patients with metastatic castration-resistant prostate cancer (TheraP): a randomised, open-label, phase 2 trial. Lancet.

[7] Hofman MS, Violet J, Hicks RJ (2018). [177Lu]-PSMA-617 radionuclide treatment in patients with metastatic castration-resistant prostate cancer (LuPSMA trial): a single-centre, single-arm, phase 2 study. Lancet Oncol.

[8] Yadav MP, Ballal S, Sahoo RK, Dwivedi SN, Bal C (2019). Radioligand Therapy With 177 Lu-PSMA for Metastatic Castration-Resistant Prostate Cancer: A Systematic Review and Meta-Analysis. Am J Roentgenol.

[9] Fanti S, Hadaschik B, Herrmann K (2020). Proposal for systemic-therapy response-assessment criteria at the time of PSMA PET/CT imaging: The PSMA PET progression criteria. J Nucl Med.

[10] Fanti S, Goffin K, Hadaschik BA Consensus statements on PSMA PET/CT response assessment criteria in prostate cancer. Eur J Nucl Med Mol Imaging. 2020.

[11] Seifert R, Herrmann K, Kleesiek J (2020). Semi-automatically quantified tumor volume using Ga-68-PSMA-11-PET as biomarker for survival in patients with advanced prostate cancer. J Nucl Med.

[12] Seifert R, Kessel K, Schlack K PSMA PET total tumor volume predicts outcome of patients with advanced prostate cancer receiving [177Lu]Lu-PSMA-617 radioligand therapy in a bicentric analysis. Eur J Nucl Med Mol Imaging. 2020.

[13] Gafita A, Bieth M, Krönke M (2019). qPSMA: Semiautomatic Software for Whole-Body Tumor Burden Assessment in Prostate Cancer Using 68 Ga-PSMA11 PET/CT. J Nucl Med.

[14] Schmuck S, Von Klot CA, Henkenberens C (2017). Initial experience with volumetric 68Ga-PSMA I&T PET/CT for assessment of whole-body tumor burden as a quantitative imaging biomarker in patients with prostate cancer. J Nucl Med.

[15] Grubmüller B, Senn D, Kramer G (2019). Response assessment using 68 Ga-PSMA ligand PET in patients undergoing 177 Lu-PSMA radioligand therapy for metastatic castration-resistant prostate cancer. Eur J Nucl Med Mol Imaging.

[16] Seifert R, Seitzer K, Herrmann K (2020). Analysis of PSMA expression and outcome in patients with advanced Prostate Cancer receiving 177 Lu-PSMA-617 Radioligand Therapy. Theranostics.

[17] Grubmüller B, Senn D, Kramer G (2019). Response assessment using 68Ga-PSMA ligand PET in patients undergoing 177Lu-PSMA radioligand therapy for metastatic castration-resistant prostate cancer. Eur J Nucl Med Mol Imaging.

[18] Eisenhauer EA, Therasse P, Bogaerts J (2009). New response evaluation criteria in solid tumours: Revised RECIST guideline (version 1.1). Eur J Cancer.

[19] Joo Hyun O, Lodge MA, Wahl RL (2016). Practical percist: A simplified guide to PET response criteria in solid tumors 1.0. Radiology.

[20] Pollard JH, Raman C, Zakharia Y (2020). Quantitative Test-Retest Measurement of 68Ga-PSMA-HBED-CC in Tumor and Normal Tissue. J Nucl Med.

[21] R Core Team (2018). R: A Language and Environment for Statistical Computing [Internet]. R Foundation for Statistical Computing, Vienna, Austria.

[22] Hofman MS, Emmett L, Sandhu SK (2020). TheraP: A randomised phase II trial of 177 Lu-PSMA-617 (LuPSMA) theranostic versus cabazitaxel in metastatic castration resistant prostate cancer (mCRPC) progressing after docetaxel: Initial results (ANZUP protocol 1603). J Clin Oncol.

[23] de Bono J, Mateo J, Fizazi K (2020). Olaparib for Metastatic Castration-Resistant Prostate Cancer. N Engl J Med.

[24] Bakht MK, Derecichei I, Li Y (2019). Neuroendocrine differentiation of prostate cancer leads to PSMA suppression. Endocr Relat Cancer.

[25] Yuan TC, Veeramani S, Lin MF (2007). Neuroendocrine-like prostate cancer cells: Neuroendocrine transdifferentiation of prostate adenocarcinoma cells. Endocr Relat Cancer.

