# Supplementary material for: Total tumor volume reduction and low PSMA expression in patients receiving Lu-PSMA therapy
Source: Theranostics. 2021 Jul 13;11(17):8143–51. doi: 10.7150/thno.60222 (PMC8344008; doi:10.7150/thno.60222)

Supporting information

Supplementary Figure 1 – Martingale analysis of total cohort

Martingale residuals of PSMA PET derived baseline parameters and PSMA-TV reduction after two cycles of Lu-PSMA. Metrics were log transformed for Cox regression.

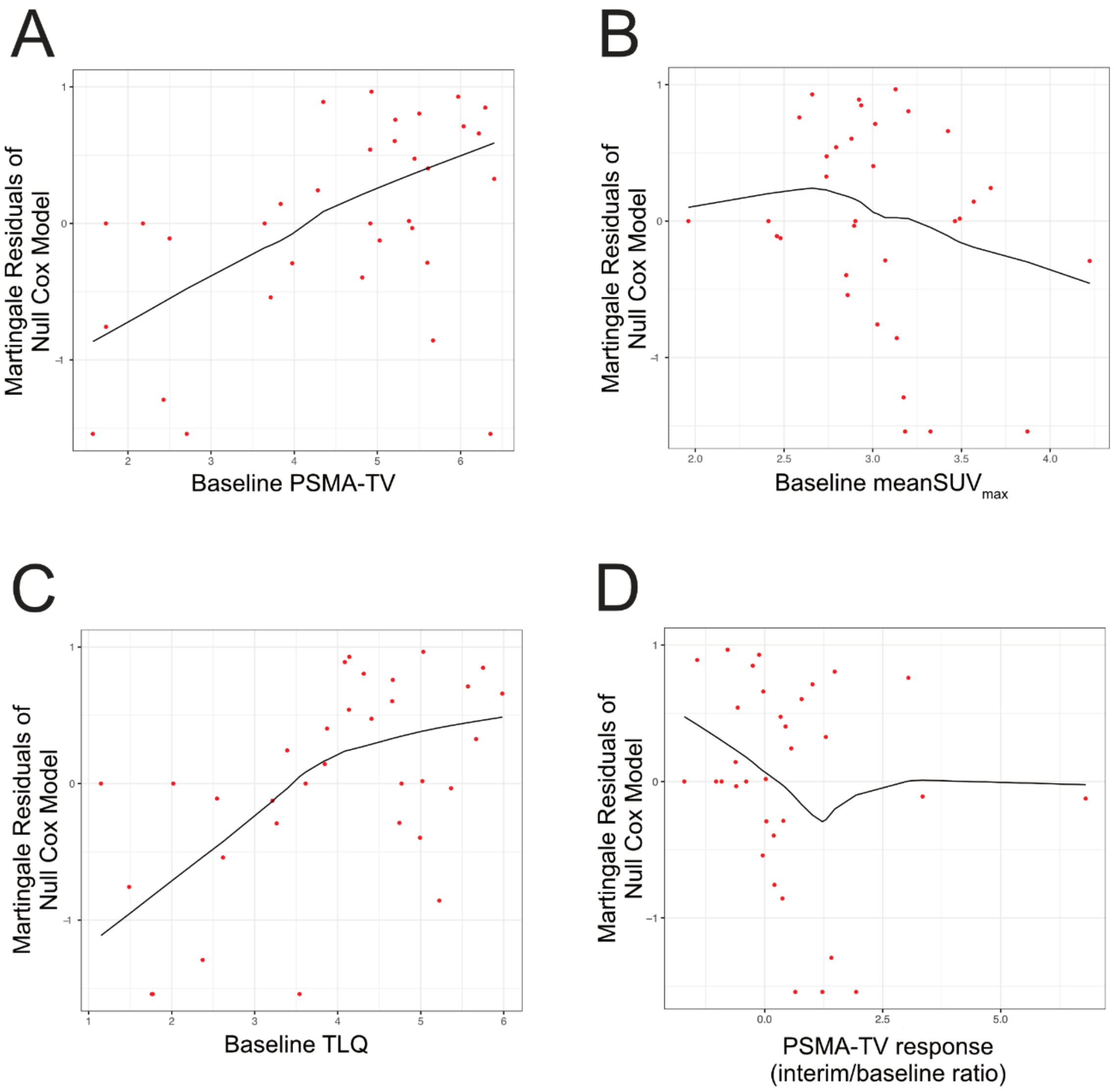

Supplement: Supplementary file 1 — Supplementary figure. [file thnov11p8143s1.pdf]
